# Supplementary material for: The role of problem-solving skills in the prevention of suicidal behaviors: A systematic review and meta-analysis
Source: PLoS One. 2023 Oct 31;18(10):e0293620. doi: 10.1371/journal.pone.0293620 (PMC10617726; doi:10.1371/journal.pone.0293620)
Supplement: S1 Table — (DOCX) [file pone.0293620.s002.docx]

**S1 Table:** Search strategies

| **Databases, time of searches, and search terms** | **Hits** |
| --- | --- |
| **PubMed to August 16, 2023** |  |
| #1 problem solving[MeSH Terms] | 30,314 |
| #2 problem solving[Text Word] | 42,603 |
| #3 coping skills[Text Word] | 3,983 |
| #4 resilience skills[Text Word] | 74 |
| #5 solution-oriented skills[Text Word] | 11 |
| #6 cognitive flexibility[Text Word] | 4,657 |
| #7 decision-making abilities[Text Word] | 382 |
| #8 thinking[MeSH Terms] | 321,794 |
| #9 critical thinking[Text Word] | 5,126 |
| #10 creativity[MeSH Terms] | 7,818 |
| #11 creative thinking[Text Word] | 860 |
| #12 analytical thinking[Text Word] | 189 |
| #13 (#1 OR #2 OR #3 OR #4 OR #5 OR #6 OR #7 OR #8 OR #9 OR #10 OR #11 OR #12) | **347,595** |
| #14 suicide[MeSH Terms] | 74,888 |
| #15 suicide[Text Word] | 99,652 |
| #16 suicidal[Text Word] | 39,933 |
| #17 suicidality[Text Word] | 9,121 |
| #18 (#14 OR #15 OR #16 OR #17) | **113,846** |
| #19 (#13 AND #18) | **2,805** |
| **Web of Science to August 16, 2023** |  |
| #1 TS=problem solving | 898,629 |
| #2 TS=coping skills | 13,549 |
| #3 TS=resilience skills | 4,993 |
| #4 TS=solution-oriented skills | 40 |
| #5 TS=cognitive flexibility | 14,188 |
| #6 TS=decision-making abilities | 32,190 |
| #7 TS=critical thinking | 68,010 |
| #8 TS=creative thinking | 18,467 |
| #9 TS= analytical thinking | 10,108 |
| #10 (#1 OR #2 OR #3 OR #4 OR #5 OR #6 OR #7 OR #8 OR #9) | **1,041,296** |
| #11 TS=suicide | 107,036 |
| #12 TS=suicidal | 46,113 |
| #13 TS=suicidality | 11,201 |
| #14 (#11 OR #12 OR #13) | **131,883** |
| #15 (#10 AND #14) | **1,775** |
| **Scopus to August 16, 2023** |  |
| #1 TITLE-ABS-KEY(problem solving) | 678,034 |
| #2 TITLE-ABS-KEY(coping skills) | 14,589 |
| #3 TITLE-ABS-KEY(resilience skills) | 5,637 |
| #4 TITLE-ABS-KEY(solution-oriented skills) | 63 |
| #5 TITLE-ABS-KEY(cognitive flexibility) | 14,680 |
| #6 TITLE-ABS-KEY(decision-making abilities) | 51,374 |
| #7 TITLE-ABS-KEY(critical thinking) | 49,897 |
| #8 TITLE-ABS-KEY(creative thinking) | 18,195 |
| #9 TITLE-ABS-KEY(analytical thinking) | 6,804 |
| #10 (#1 OR #2 OR #3 OR #4 OR #5 OR #6 OR #7 OR #8 OR #9) | **818,364** |
| #11 TITLE-ABS-KEY(suicide) | 151,036 |
| #12 TITLE-ABS-KEY(suicidal) | 67,945 |
| #13 TITLE-ABS-KEY(suicidality) | 11,218 |
| #14 (#11 OR #12 OR #13) | **178,523** |
| #15 (#10 AND #14) | **2,133** |
| **Total** | **6,713** |
| **Duplicates** | 2,458 |
| **Final** | **4,255** |
