# Supplementary material for: The role of problem-solving skills in the prevention of suicidal behaviors: A systematic review and meta-analysis
Source: PLoS One. 2023 Oct 31;18(10):e0293620. doi: 10.1371/journal.pone.0293620 (PMC10617726; doi:10.1371/journal.pone.0293620)
Supplement: S2 Table — (DOCX) [file pone.0293620.s003.docx]

**S2 Table:** The detailed risk of bias assessment

| 1st author, year | S1 | S2 | S3 | S4 | C1 | E1 | E2 | E3 | Total score | Quality |
| --- | --- | --- | --- | --- | --- | --- | --- | --- | --- | --- |
| Observational studies |  |  |  |  |  |  |  |  |  |  |
| Akbari, 2015 | 1 | 1 | 1 | 1 | 2 | 0 | 1 | 1 | ★★★★★★★ | High |
| Biggam, 1999 | 1 | 1 | 0 | 1 | 0 | 0 | 1 | 1 | ★★★★★ | Low |
| Burke, 2016 | 1 | 1 | 0 | 1 | 2 | 0 | 1 | 1 | ★★★★★★★ | High |
| Dieserud, 2002 | 1 | 1 | 1 | 1 | 2 | 0 | 1 | 1 | ★★★★★★★ | High |
| Donald, 2006 | 1 | 1 | 1 | 1 | 2 | 0 | 1 | 1 | ★★★★★★★ | High |
| Eidhin, 2002 | 1 | 1 | 0 | 1 | 0 | 0 | 1 | 1 | ★★★★★ | Low |
| Foroughipour, 2013 | 1 | 1 | 0 | 1 | 0 | 1 | 1 | 1 | ★★★★★★ | Low |
| Fried, 2013 | 1 | 1 | 1 | 1 | 0 | 0 | 1 | 1 | ★★★★★★ | Low |
| Gururaj, 2004 | 1 | 1 | 1 | 1 | 0 | 0 | 1 | 1 | ★★★★★★ | Low |
| Howat, 2002 | 1 | 1 | 1 | 1 | 0 | 0 | 1 | 1 | ★★★★★★ | Low |
| Kaviani, 2004 | 1 | 1 | 1 | 1 | 0 | 0 | 1 | 1 | ★★★★★★ | Low |
| Kidd, 2007 | 1 | 1 | 1 | 1 | 1 | 1 | 1 | 1 | ★★★★★★★★ | High |
| Lannoy, 2021 | 1 | 1 | 1 | 1 | 2 | 0 | 1 | 1 | ★★★★★★★★ | High |
| Li, 2012 | 1 | 1 | 1 | 1 | 0 | 1 | 1 | 1 | ★★★★★★★ | High |
| Linda, 2012 | 1 | 1 | 1 | 1 | 0 | 0 | 1 | 1 | ★★★★★★ | Low |
| Mostafavi Rad, 2012 | 1 | 1 | 1 | 1 | 0 | 0 | 1 | 1 | ★★★★★★ | Low |
| Nezu, 2017 | 1 | 1 | 1 | 1 | 0 | 1 | 1 | 1 | ★★★★★★★ | High |
| Pollock, 2001 | 1 | 1 | 0 | 1 | 0 | 0 | 1 | 1 | ★★★★★ | Low |
| Roskar, 2007 | 1 | 1 | 1 | 1 | 0 | 0 | 1 | 1 | ★★★★★★ | Low |
| Sadowski, 1993 | 1 | 1 | 1 | 1 | 0 | 0 | 1 | 1 | ★★★★★★ | Low |
| Sarkisian, 2021 | 1 | 1 | 1 | 1 | 2 | 0 | 1 | 1 | ★★★★★★★ | High |
| Shelef, 2014 | 1 | 1 | 1 | 1 | 0 | 0 | 1 | 1 | ★★★★★★ | Low |
| Sugawara, 2012 | 1 | 1 | 1 | 1 | 2 | 0 | 1 | 1 | ★★★★★★★★ | High |
| Tang, 2015 | 1 | 1 | 1 | 1 | 2 | 0 | 1 | 1 | ★★★★★★★★ | High |
| Randomized control trials | **T** | **B** | **E** | **B1** | **B2** | **B3** | **V** | **I** | **Total score** | **Quality** |
| Angora, 2022 | 0 | 0 | 1 | 1 | 0 | 0 | 1 | 1 | ★★★★ | Low |
| Eskin, 2008 | 1 | 1 | 0 | 1 | 0 | 0 | 1 | 1 | ★★★★★ | Low |
| Fitzpatrick, 2005 | 1 | 0 | 1 | 1 | 0 | 0 | 0 | 1 | ★★★★ | Low |
| Unützer, 2006 | 1 | 1 | 1 | 1 | 1 | 0 | 1 | 1 | ★★★★★★★ | High |
| Xavier, 2019 | 1 | 1 | 1 | 1 | 1 | 0 | 1 | 1 | ★★★★★★★ | High |

**Newcastle Ottawa Statement (NOS) Manual**

**S1**: Selection: An adequate case definition ★

**S2**: Selection: Representativeness of the cases ★

**S3**: Selection: Selection of controls ★

**S4**: Selection: Definition of controls ★

**C1**: Comparability: Comparability of cases and controls on the basis of the design or analysis ★★

**E1**: Exposure: Ascertainment of exposure ★

**E2**: Exposure: Same method of ascertainment for cases and controls ★

**E3**: Exposure: Non-Response rate ★

**Delphi List**

**T**: Treatment allocation: (a) Was a method of randomization performed? (b) Was the treatment allocation concealed? ★★

**B**: Were the groups similar at baseline regarding the most important prognostic indicators? ★

**E**: Were the eligibility criteria specified? ★

**B1**: Was the outcome assessor blinded? ★

**B2**: Was the care provider blinded? ★

**B3**: Was the patient blinded? ★

**V**: Were point estimates and measures of variability presented for the primary outcome measures? ★

**I**: Did the analysis include an intention-to-treat analysis? ★
