# Supplementary material for: The role of problem-solving skills in the prevention of suicidal behaviors: A systematic review and meta-analysis
Source: PLoS One. 2023 Oct 31;18(10):e0293620. doi: 10.1371/journal.pone.0293620 (PMC10617726; doi:10.1371/journal.pone.0293620)
Supplement: Attachment — Submitted filename: Response to Reviewers.docx [file pone.0293620.s005.docx]

**Additional Editor Comments**

Thank you for submitting your manuscript to PLOS ONE. After careful consideration, we feel that it has merit but does not fully meet PLOS ONE’s publication criteria as it currently stands. Therefore, we invite you to submit a revised version of the manuscript that addresses the points raised during the review process.

Please revise the interpretation of the pooled estimate as the statistical significance should be not emphasized in the meta-analysis.

**Answer**: We omitted the terms "significant" and "significantly" from both the abstract and the main results and discussion sections, where we highlighted the outcomes of the meta-analysis.

Limitation section and a recommendation section are missing.

**Answer**: These sections were added to the end of the Discussion section.

Is it only search strategy, "The following keywords were searched: (problem solving OR problem-solving) AND (suicide OR suicidal OR suicidality)"? If yes, then the search is very narrow and may be no longer systematic research. Describe the details of the search like if you used the MES heading and keywords both. The search strategies should be provided in a supplementary file.

**Answer**: We have diligently expanded upon our search strategy and incorporated the latest search findings up until August 16, 2023. Our search encompassed a range of pertinent keywords, including terms such as (problem solving or coping skills or resilience skills or solution-oriented skills or cognitive flexibility or decision-making abilities or critical thinking or creative thinking or analytical thinking) and (suicide or suicidal or suicidality). It is noteworthy that both MeSH terms and keywords were thoughtfully integrated into our comprehensive search approach. Fortuitously, the implementation of the new search strategy yielded two additional studies, culminating in a total of 29 studies now included in our analysis. The detailed outline of our search strategies can be found in the supplementary file, providing a clear reference for the methods employed.

EMBASE and other databases were not covered which may be the potential limitation of the review.

**Answer**: Regrettably, we did not have access to the EMBASE database during our research phase. Nonetheless, a silver lining exists as both the EMBASE and Scopus databases fall under the umbrella of Elsevier Publications. This interconnection ensures that a substantial portion of the resources cataloged in EMBASE are encompassed within the scope of Scopus. Notably, Mann et al. conducted a thorough investigation in 2016 [1] aimed at evaluating coverage, overlap, and distinctive contributions of EMBASE and Scopus. Their findings revealed that 32 of the 34 studies included were sourced from either the EMBASE or Scopus searches. These results offer a compelling insight into the interplay between these databases.

[1] Mann M, Hood K, Trubey R, Powell C. A tale of two databases: a comparison of Embase versus Scopus. In: Challenges to evidence-based health care and Cochrane. Abstracts of the 24th Cochrane Colloquium; 2016 23-27 Oct; Seoul, Korea. John Wiley & Sons; 2016.

How was the RCT incorporated in this study along with other designs? Randomized clinical trials (RCT) addressing the association between problem-solving skills and suicide behaviors" RCT does not incorporate association. Use subheadings while reporting the results.

**Answer**: We acknowledge the distinct nature of observational studies and randomized controlled trials (RCTs), and we concur that they should be treated as separate entities when consolidating data. To address this valid concern, we have been diligent in our approach, ensuring that each study type receives the due attention it warrants. In the "Methodological Quality" subsection within the Methods section of our manuscript, we have clearly outlined our strategy. Specifically, we employed the Newcastle-Ottawa Scale (NOS) to assess the quality of observational studies and utilized the Delphi List to evaluate the quality of RCTs. Furthermore, we have taken into account the differentiation between the outcomes of observational studies and RCTs in our results. As a result, we have represented the results of observational studies in Figures 2 & 4 and RCTs in Figures 3 & 5. We deeply value your insights and have diligently addressed the issues raised to ensure the accuracy and clarity of our manuscript.

"Problem-solving skills" how this intervention was measured throughout the study?

**Answer**: In our study, the assessment of “problem-solving skills” was carried out through the utilization of dedicated questionnaires that have been developed and validated for the purpose of evaluating problem-solving abilities. These questionnaires have been widely recognized and accepted within the field as reliable tools for assessing the proficiency of individuals in problem-solving. We addressed this matter by incorporating an explanatory note at the outset of the Methods section to provide clarification. Additionally, a comprehensive listing of these questionnaires is available in Table 1 for reference.

Inclusion and exclusion are missing.

**Answer**: The inclusion and exclusion criteria for our meta-analysis were defined using the PICOS framework, which encompasses Population, Intervention/Exposure, Comparison, Outcome, and Study design characteristics. These criteria were systematically applied to identify relevant studies for our analysis. We incorporated these criteria at the outset of the Methods section to provide a clear understanding of our study's criteria for study selection.

“Control: In cases where studies categorized problem-solving skills, we examined the possibility of suicidal behaviors in individuals with high problem-solving skills compared to those with low problem solving skills as a control group.” What does it mean? Specify who is the comparator. If the intervention is problem-solving skills then the comparator should be something else, maybe not having the skill.

**Answer**: Certainly, I appreciate your feedback. In our study, we adopted two distinct approaches to investigate the association between problem-solving skills and suicidal behaviors.

Firstly, in cases where studies categorized individuals based on their problem-solving skills (high vs. low), we examined the odds of suicidal behaviors in those with high problem-solving skills compared to those with low problem-solving skills (reference group). This allowed us to assess the impact of varying problem-solving skill levels on suicidal behaviors.

Secondly, we encountered studies that measured the mean problem-solving skill scores among individuals with and without suicidal behaviors. In these cases, we calculated the standardized mean difference (SMD) of the problem-solving skill scores between these two groups. This approach enabled us to explore the magnitude of difference in problem-solving abilities between individuals exhibiting suicidal behaviors and those who did not.

To present these distinct analyses accurately, we reported the pooled odds ratios for the first approach and the SMD values for the second approach separately. By employing these two different methodologies, we aimed to comprehensively capture the relationship between problem-solving skills and suicidal behaviors, providing a more nuanced understanding of their interplay. We clarified these points further in revised manuscript for better clarity and transparency.

“In cases where studies reported the mean score of problem-solving skills, we examined the standard mean difference between those with and without suicidal behaviors.” SMD of which groups? Does it a suicidal idea vs no suicidal ideation? If yes, then I think, it is a serious gap in the planning.

**Answer**: Thank you for your feedback. We have taken your current comments into consideration and addressed the concerns raised in response to your previous comment. We are committed to ensuring the clarity and accuracy of our manuscript, and we have incorporated the suggestions and considerations discussed earlier. We appreciate your guidance and are dedicated to producing a thorough and well-structured manuscript that adheres to the highest standards of research integrity.

Cross-sectional study and RCT cannot be pooled in same study and should have followed different tools for doing the risk of bias and certainty of the evidence. Meta-analysis should not be pooling apple and orange.

**Answer**: We appreciate the editor's observation. We completely understand the importance of maintaining methodological rigor when conducting a meta-analysis. We agree that observational studies and randomized controlled trials (RCTs) are distinct in nature and should be treated as separate entities when pooling data. To address this concern, we would like to highlight that we were meticulous in our approach to ensure that each study type received appropriate attention. Specifically, under the "Methodological Quality" subsection in the Methods section of our manuscript, we clearly delineated our approach. For observational studies, we employed the Newcastle-Ottawa Scale (NOS) to assess their quality, while for RCTs, we used the Delphi List to evaluate their quality. This approach enabled us to rigorously evaluate the risk of bias and the certainty of evidence for each study type, ensuring that the distinct characteristics of observational studies and RCTs were appropriately considered. Certainly, we recognize the importance of maintaining a clear distinction between the results of observational studies and RCTs within our figures. To address this, the results of observational studies are depicted separately in Figures 2 & 4 and that of and RCTs in Figures 3 and 5.

“This meta-analysis was conducted to determine to what extent problem-solving skills can prevent suicidal behaviors”. If this is the objective of this review and then I think this review failed to achieve the objective.

**Answer**: Thank you for your feedback. We appreciate your insights, and we have carefully considered your comments. In response to your concern, we have revised the objective of the meta-analysis to ensure clarity and alignment with the study's findings. The modified objective now reads: “This meta-analysis was conducted to assess the association between problem-solving skills and suicidal behaviors and elucidate the potential role of problem-solving skills in influencing the occurrence of suicidal behaviors.”

We believe that this modified objective better captures the focus and outcomes of our study, and we are grateful for your input in refining our work.
