# Supplementary material for: The role of problem-solving skills in the prevention of suicidal behaviors: A systematic review and meta-analysis
Source: PLoS One. 2023 Oct 31;18(10):e0293620. doi: 10.1371/journal.pone.0293620 (PMC10617726; doi:10.1371/journal.pone.0293620)
Supplement: Attachment — Submitted filename: Response to Reviewers.docx [file pone.0293620.s006.docx]

**Reviewer #3**

Thanks for giving me the opportunity to review this manuscript. After careful revision of this manuscript, I suggest the following minor edits.

1. Methods: the heterogeneity and publication bias section should be reported below the Data synthesis section.

**Response**: Thank you for your valuable feedback and for your careful review of our manuscript. We have carefully considered your suggestion regarding the placement of the "Heterogeneity and publication bias" section in our manuscript. Following your recommendation, we have moved this section below the "Data synthesis" section to enhance the logical flow and organization of our paper.

2. Authors are suggested to report the detailed risk of bias assessment in the supplementary file.

**Response**: Regarding your suggestion to report the detailed risk of bias assessment in the supplementary file, we have taken this recommendation into account. We have included a comprehensive risk of bias assessment in the supplementary material, providing a detailed and transparent account of our evaluation process.

3. The recommendation section should be merged with the discussion section without mentioning the heading "Recommendation"

**Response**: In accordance with your recommendation, we have removed the heading "Recommendation" and integrated the content seamlessly into the broader "Discussion" section.
